# Effect of pH of amine fluoride containing toothpastes on enamel remineralization in vitro

**DOI:** 10.1186/1472-6831-7-14

**Published:** 2007-10-17

**Authors:** Wolfgang H Arnold, Anabel Haase, Julia Hacklaender, Zeno Gintner, Jolan Bánóczy, Peter Gaengler

**Affiliations:** 1 Faculty of Dental Medicine, University of Witten/Herdecke, Witten, Germany; 2 Faculty of Dentistry, Semmelweis University, Budapest, Hungary

## Abstract

**Background:**

One of the important factors of the demineralization and remineralization equilibrium of enamel is the pH of the surrounding solutions. Effort has been laid in the formulation of different fluoride compounds and the fluoride content in toothpastes but much less is known about the influence of the pH of the toothpastes on their effectiveness. It was therefore the aim of this study to investigate the influence of different pH levels on enamel remineralization in an in vitro experiment using polarization light microscopy and EDX quantitative element analysis.

**Methods:**

A 5 × 5 mm window on the enamel surface of 40 caries free extracted human premolars was demineralized in a hydroxyethylcellulose solution at pH 4.8. The teeth were divided into 8 groups and the lower half of the window was covered with varnish serving as control. Each group was then immersed in toothpaste slurry containing amine fluoride (1400 ppm) at pH 4.1, 4.5, 5.1 and 6.9 or control toothpaste slurry without fluoride at pH 4.3, 4.7, 5.3 and 7.0. Serial sections were cut through the lesions and investigated with polarization light microscopy and quantitative EDX element analysis.

**Results:**

The PLM results showed a decreased porous volume of the body of the lesion after incubation with fluoridated toothpaste at pH 4.53 and 5.16. No differences between the experimental window and the control window were found in the other groups. The quantitative element analysis showed no differences in the element content of any of the groups.

**Conclusion:**

From the results it can be concluded that slightly acidified fluoridated dentifrices may have a certain positive effect on enamel remineralization.

## Background

Dental caries progression or reversal depends upon the balance between demineralization and remineralization [1]. This balance is depended from several factors e.g. salivary Ca and P concentration, bioavailability of fluoride and pH. Remineralization occurs when the pH raises and Ca and P from saliva together with fluoride are forming new hydroxyapatite crystals on the enamel surface and the body of the lesion [2,3]. Mineral loss of incipient caries lesions is inversely proportional to the degree of saturation of Ca and P ions and the pH of the solution The critical pH range for demineralization and remineralization is between 4.3 and 5.0 where lesions with well defined surface layers occur whereas at pH levels around 6.0 no surface layers are forming [4].

Fluoride plays an important role in the remineralization process. Although a dose-response effect of fluoride enhancing enamel remineralization has been found [5,6] also small amounts of fluoride (<1 ppm) act on remineralization [7,8]. Demineralization and remineralization experiments have shown that fluoride enhances mineral uptake during continuous remineralization [9]. It is now acknowledged that fluoride acts as catalyst and influences reaction rates with dissolution and transformation of various calcium phosphate minerals. At low fluoride levels (<0.1 ppm) calcium uptake during remineralization is enhanced, while at this concentration no effect of fluoride on enamel demineralization is observed [10]. Below the critical pH hydroxyapatite is dissolved, but the released mineral ions could be reprecipitated as fluorapatite which is less soluble and may provide additional protection onto the apatite crystals. Even at a physiological pH fluorapatite precipitation is greater than that of hydroxyapatite also at low fluoride levels [11]. However, TenCate and Duijsters [12,13] showed that fluorapatite per se did not affect the overall mineral loss in enamel but calcium fluoride which is more effective in inhibiting enamel demineralization than fluorapatite.

Most of the studies concerning the effects of fluoridated toothpastes on enamel remineralization have been carried out with respect to the amount of fluoride [6,14-16] or different fluoride compounds [17-20]. Only little attention has been paid to the influence of different pH values of fluoridated toothpastes on enamel remineralization [21,22]. From a physico-chemical point of view it seems to be reasonable to investigate the influence of fluoride toothpastes on enamel remineralization under various pH conditions.

## Methods

Forty for orthodontical reasons extracted caries free premolars were covered with varnish leaving a 5 × 5 mm window and randomly divided into 8 groups of 5 teeth in each group. They were kept in a demineralizing gel (hydroxyethylcellulose) at pH 4.95 for 50 days. After demineralization the lower half of the window was also covered with varnish serving as positive control. Each group was then incubated in amine fluoride (1400 ppm) containing toothpaste slurries and control slurries without amine fluoride at different pH levels for 48 hours which is equivalent to 2 years tooth brushing 2 times for 2 minutes per day [23]. For the slurries 40 cm^3 ^of experimental toothpaste was mixed with 160 ml dist. water. The pH of the slurries was checked prior to the incubation of the teeth, after 24 hours and after 48 hours prior to the termination of the incubation. Incubation media are summarized in Table 1.

**Table 1:** Test and control slurries with different pH levels.

Group	pH
Group 1 with amine fluorid 1400 ppm	4,1
Group 2 with amine fluorid 1400 ppm	4,5
Group 3 with amine fluorid 1400 ppm	5,1
Group 4 with amine fluorid 1400 ppm	6,9
Group 5 without amine fluorid (control)	4,3
Group 6 without amine fluorid (control)	4,7
Group 7 without amine fluorid (control)	5,3
Group 8 without amine fluorid (control)	7,0

After treatment with slurries the teeth were embedded in Technovit 9100 (Kulzer, Germany) and serial sections through the lesions with a thickness of 80 μm were cut using a saw microtome (Leica 1600, Germany). All sections were investigated with polarization light microscopy (PLM) and three central sections of each lesion were categorized according to their morphological appearance and to each category was a numerical index number assigned into: not present (1), single porosities (2), interrupted band (3), inhomogeneous (4), completely homogeneous (5), more than 60 μm depth (6). The numerical values were statistically compared using the nonparametric Mann-Whitney test.

Three sections of each lesion were then coated with carbon and examined with a scanning electron microscope (Philips XL 30 FEG) at 20 kV using the backscattered electron detector In each experimental and control window of the different teeth 3 spot measurements (spot size 2 nm) were carried out on the enamel surface, within the body of the lesion and sound enamel, resulting in a total number of 9 measuring points per window. Element content in weight % of Ca, P, C, and F was measured with energy dispersive X-ray analysis (EDX) with a S-UTW detector (EDAX INC, Mahwah, NJ, USA). The count rate of the EDX detector was between 1800 and 2000 counts per second with a dead time of 30%. Measuring time was 30 s (live seconds) with a resolution of 135.8 eV and an amplification time of 100 μs. Line scans through the lesions were made at 256 points with a dwell time of 1000 ms and amplification time of 100 ms. The values of the spot measurements were statistically evaluated using the nonparametric ANOVA test for repeated measurements.

## Results

### Lesion morphology

**Figure 1:**
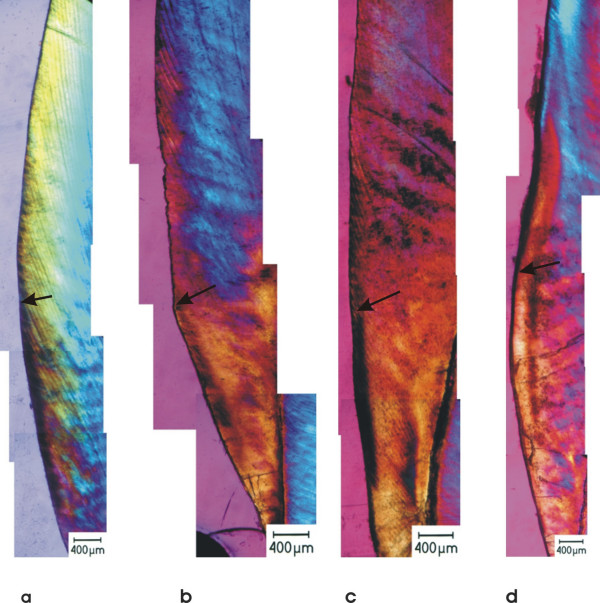
**Polarization light microscopy of experimental lesions**. Experimental caries-like lesions after treatment with fluoridated toothpaste at different pH levels. a) pH 4.1; the upper experimental window shows an interrupted band. b) pH 4.5; the upper experimental window shows single porosities. c) pH 5.1; the upper experimental window shows an inhomogeneous lesion. d) pH 6.9; no difference can be seen between the upper experimental window and the lower control window.

**Figure 2:**
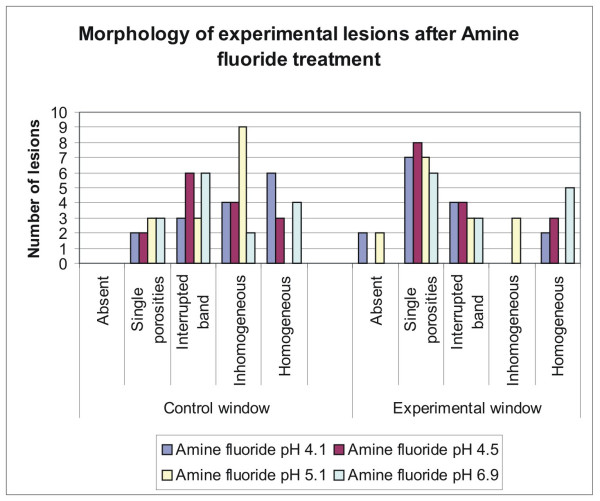
**Quantitative distribution of the different experimental lesions**. Quantitative distribution of lesions according to their morphological appearance in control and experimental windows after fluoride application at different pH levels. There is a clear shift of the lesion morphology towards the less expressed lesions in the experimental windows.

**Figure 3:**
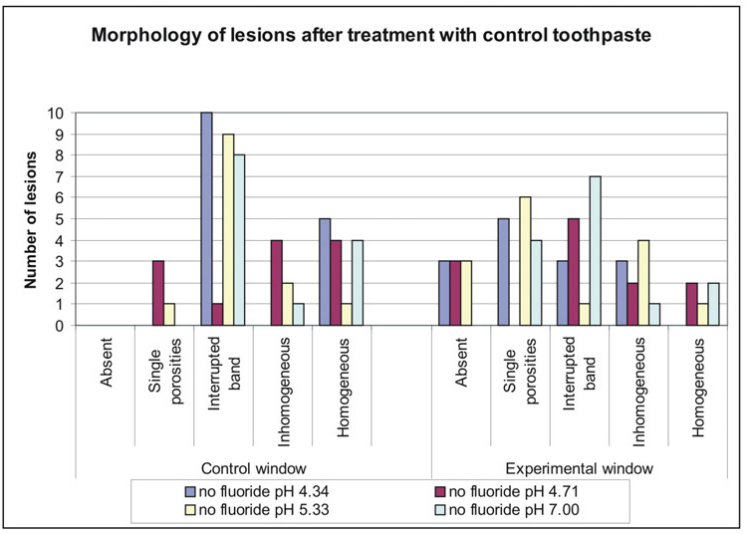
**Quantitative distribution of the different control lesions**. Quantitative distribution of lesions according to their morphological appearance in control and experimental windows after application of control toothpaste at different pH levels. The different morphologies are more or less equally distributed.

Morphological analysis of the sections with PLM revealed variable expression of the lesions after incubation at different pH values (Fig. 1). They were mainly expressed as inhomogeneous or homogeneous lesions, interrupted bands and as single porosities. In the experimental windows most of the lesions were expressed as single porosities and interrupted bands after treatment with fluoride Fig. 2), whereas with non fluoridated toothpastes the experimental lesions were mostly inhomogeneous or homogeneous Fig. 3). Significant differences in the lesion morphology between the control window and the experimental window were found in group 2 (pH 4.53; p = 0.032) and group 3 (pH 5.16; p = 0.014) after fluoride treatment. In the experimental windows a larger number of lesions with single porosities or interrupted bands was found than in the control windows. In all other groups no significant difference was found (p > 0.05).

### EDX analysis

EDX element analysis showed no statistically significant differences in the element content for Ca, P, C and F in the body of the lesion and the superficial enamel layer. The mean element content in the body of the lesion of Ca was between 33 wt% and 41.9 wt%, for P the content was between 16.6 wt% and 19.9 wt%, for C it was between 6.5 wt% and 12.6 wt% and for F it was between 0.27 wt% and 0.68 wt%. All results are summarized in Table 2 for the body of the lesion and Table 3 for the superficial enamel layer.

**Table 2:** Mean element content in the body of the lesion of control and experimental window.

	Experimental window				Control window			
	
Group/element	Ca	P	C	F	Ca	P	C	F
Amine fluoride pH 4.1	34.1 ± 3.6	18.3 ± 1.3	9.3 ± 2.5	0.33 ± 0.22	34.2 ± 3.6	18.5 ± 1.1	8.5 ± 2.0	0.34 ± 0.25
Amine fluoride pH 4.5	34.5 ± 5.5	19.0 ± 2.7	8.5 ± 5.4	0.47 ± 0.4	33.5 ± 6.0	18.3 ± 1.6	9.1 ± 2.7	0.42 ± 0.3
Amine fluoride pH 5.1	35.5 ± 4.4	19.1 ± 1.4	7.4 ± 3.8	0.48 ± 0.39	35.0 ± 5.1	18.9 ± 1.6	8.0 ± 3.6	0.57 ± 0.45
Amine fluoride pH 6.9	34.6 ± 5.5	18.6 ± 2.2	9.4 ± 7.4	0.4 ± 0.26	33.7 ± 6.4	18.5 ± 2.9	8.8 ± 9.6	0.57 ± 3.4
no fluoride pH 4.3	34.8 ± 5.5	18.9 ± 2.1	8.0 ± 6.6	0.35 ± 0.28	35.1 ± 4.3	18.9 ± 1.2	8.2 ± 4.5	0.41 ± 0.33
no fluoride pH 4.7	35.45 ± 5.8	18.7 ± 2.1	9.5 ± 4.5	0.3 ± 0.28	35.1 ± 3.2	18.8 ± 1.1	7.8 ± 3.1	0.35 ± 0.27
no fluoride pH 5.3	35.0 ± 4.7	18.7 ± 1.6	9.0 ± 4.1	0.34 ± 0.25	34.9 ± 3.9	18.6 ± 1.2	9.6 ± 2.2	0.46 ± 0.32
no fluoride pH 7.0	41.9 ± 19.8	17.4 ± 4.6	5.5 ± 4.8	0.28 ± 0.3	40.3 ± 20.0	16.6 ± 4.3	7.7 ± 4.0	0.38 ± 0.31

**Table 3:** Mean element content in the superficial enamel layer of control and experimental window.

	Experimental window				Control window			
	
Group/element	Ca	P	C	F	Ca	P	C	F
Amine fluoride pH 4.1	36.6 ± 4.5	18.4 ± 1.5	9.6 ± 2.8	0.42 ± 0.43	35.0 ± 5.5	19.0 ± 1.6	8.4 ± 2.9	0.3 ± 0.31
Amine fluoride pH 4.5	37.4 ± 3.3	19.9 ± 1.2	6.5 ± 3.3	0.36 ± 0.28	34.9 ± 6.1	19.0 ± 1.5	7.6 ± 2.9	0.45 ± 0.34
Amine fluoride pH 5.1	35.9 ± 4.8	19.1 ± 1.1	7.5 ± 3.5	0.49 ± 0.63	36.5 ± 5.6	19.4 ± 1.8	7.6 ± 4.2	0.58 ± 0.41
Amine fluoride pH 6.9	35.3 ± 3.7	19.0 ± 1.7	9.8 ± 7.5	0.53 ± 0.54	35.7 ± 4.6	19.4 ± 1.2	6.7 ± 4.1	0.48 ± 0.29
no fluoride pH 4.3	35.7 ± 4.6	18.9 ± 1.6	8.5 ± 4.9	0.46 ± 0.34	36.4 ± 4.9	19.5 ± 1.2	7.4 ± 4.1	0.68 ± 1.9
no fluoride pH 4.7	35.9 ± 3.1	18.9 ± 1.2	9.0 ± 3.6	0.4 ± 0.24	35.4 ± 3.5	18.7 ± 1.2	9.0 ± 3.2	0.43 ± 0.35
no fluoride pH 5.3	33.6 ± 7.0	17.9 ± 3.3	12.6 ± 10.6	0.45 ± 0.31	35.0 ± 4.4	18.6 ± 1.5	9.5 ± 4.2	0.34 ± 0.21
no fluoride pH 7.0	41.7 ± 19.8	17.2 ± 4.5	8.0 ± 5.7	0.27 ± 0.24	41.5 ± 19.6	17.0 ± 4.5	7.7 ± 7.1	0.29 ± 0.26

## Discussion

One of the main causes for enamel demineralization is undoubtedly the drop of pH below the critical point for hydroxyapatite dissolution [24]. The equilibrium between enamel demineralization and remineralization maintains an intact enamel surface [1]. At the critical pH for hydroxyapatite dissolution fluorapatite and calcium fluoride are supersaturated and may be deposited in lesion pores of enamel reducing further demineralization [11]. On the other hand, fluoride has been discussed being a catalyst for the transformation of different phosphate minerals rather than forming fluorapatite [7]. The results of this investigation support the latter as there were no differences in the element content between the experimental and control caries like lesions. Furthermore, no increased fluoride content could be determined which would be likely in fluorapatite formation.

The results of this investigation showed an increased remineralization at pH levels between 4.5 and 5.1 under the influence of amine fluoride because the porous volume of the body of the lesion was significantly reduced. Supersaturation of hydroxyapatite is limited to a pH range of 5.6–5.8 [4,11] with the consequence that hydroxyapatite formation at a lower pH would not be likely. However, in the presence of fluoride at a pH between 4.5 and 5.1 the released mineral ions could be reprecipitated as mixed fluor-hydroxyapatite enhancing remineralization of the body of the lesion and the enamel surface layer. The results of the quantitative EDX element analysis confirm the presence of hydroxyapatite in the body of the lesion and in the superficial enamel layer of both the control window and fluoride treated experimental window.

It could be argued that the application of slightly acidified toothpastes may result in erosion of the enamel surface. Erosion occurs at much lower pH levels where the solutions are undersaturated with respect to hydroxyapatite and also fluorapatite [25] and therefore, remineralization would not be possible with respect to thermodynamics. The presented results also showed no erosion of the enamel surface in any of the caries like lesions.

It has been shown that fluoride enhances mineral uptake during enamel remineralization, and inhibits mineral loss during demineralization [9,12,13]. Formation of Calcium fluoride plays an important role in the cariostatic effect of topical fluoride and is pH dependent. It is probably the major reaction product on dental hard tissues from short treatments with relatively concentrated fluoride agents and serves as fluoride reservoir [26]. Demineralization is significantly reduced below the saturation line of calcium fluoride and the formation of hydroyapatite is increased at lower pH values in the presence of calcium fluoride [11]. Calcium fluoride formation depends from pH and is less soluble at low pH values.

## Conclusion

From the results of this study it can be concluded that the pH of the dentifrices also plays an important role in their effectiveness. Slightly acidified fluoride containing dentifrices may have a certain effect on enamel remineralization.

## Competing interests

The author(s) declare that they have no competing interests.

## Authors' contributions

WHA was the supervisor of the project and responsible for the manuscript draft.

AH carried out the PLM investigations

JH carried out the EDX measurements

ZG carried out the slurry experiments and F measurements

JB supported the experiments of ZG and contributed to the manuscript draft

PG contributed to the planning of the project, evaluation of the results and writing of the manuscript

All authors have read and approved the final version of the manuscript.

## Pre-publication history

The pre-publication history for this paper can be accessed here:


